# Probiotics and infective endocarditis in patients with hereditary hemorrhagic telangiectasia: a clinical case and a review of the literature

**DOI:** 10.1186/s12879-018-2956-5

**Published:** 2018-02-01

**Authors:** Evangelo Boumis, Alessandro Capone, Vincenzo Galati, Carolina Venditti, Nicola Petrosillo

**Affiliations:** National Institute for Infectious Diseases “Lazzaro Spallanzani” IRCCS, Rome, Italy

**Keywords:** Probiotics, *Lactobacillus rhamnosus*, Infective endocarditis, Hereditary hemorrhagic telangiectasia

## Abstract

**Background:**

In the last decades, probiotics have been widely used as food supplements because of their putative beneficial health effects. They are generally considered safe but rare reports of serious infections caused by bacteria included in the definition of probiotics raise concerns on their potential pathogenic role in patients with particular predisposing factors. Patients with hereditary hemorrhagic telangiectasia (HHT) are exposed to infections because of telangiectasias and arteriovenous malformations (AVMs). We describe what is, to our knowledge, the first case of infective endocarditis (IE) caused by *Lactobacillus rhamnosus* in a patient with HHT. A systematic review of the relevant medical literature is presented.

**Case presentation:**

A patient with HHT and an aortic bioprosthesis was admitted because of prolonged fever not responding to antibiotics. The patient had a history of repeated serious infections with hospitalizations and prolonged use of antibiotics, and used to assume large amounts of different commercial products containing probiotics. Weeks before the onset of symptoms the patient had been treated with nasal packings and with surgical closure of a nasal bleeding site because of recurrent epistaxis. A diagnosis of IE of the aortic bioprosthesis was made. All blood coltures were positive for *L. rhamnosus*. The patients responded to a cycle of 6 weeks of amoxicillin/clavulanate plus gentamicin. A systematic review of IE linked to consumption of probiotics, and of infective endocarditis in patients with HHT was conducted. 10 cases of IE linked to probiotics consumption and 6 cases of IE in patients with HHT were found.

**Conclusions:**

Consumption of probiotics can pose a risk of serious infections in patients with particular predisposing factors. Patients with HHT can be considered at risk because of their predisposition to infections. Prophylaxis with antibiotics before nasal packings in patients with HHT can be considered.

**Electronic supplementary material:**

The online version of this article (10.1186/s12879-018-2956-5) contains supplementary material, which is available to authorized users.

## Background

Probiotics are described as live microbial feed supplements that beneficially affect the host animal by improving its microbial balance [1]. Many microorganisms are considered probiotics, among these bacterial species of *Bifidobacterium*, *Lactobacillus*, *Streptococcus*, *Enterococcus* and yeasts like Saccharomyces. Many of these microorganisms constitute part of the normal human flora. Lactobacilli for example are normally present in the oral cavity, ileum and colon and are the dominant microorganisms in the vagina [2]; these are also the most widely represented microorganisms in probiotics.

The use of probiotics is widespread. Traditionally, in many countries they are consumed as fermented dairy food products like yogurt. Moreover, in the last two decades their use as food supplements has greatly increased because of their putative beneficial health effects (https://www.naturalproductsinsider.com/articles/2016/09/the-new-market-profile-of-probiotics-consumption.aspx) [3]. Their use has also increased in the healthcare setting, especially among patients with impaired intestinal integrity and chronic conditions or immunosuppression, like diabetes mellitus, chronic renal failure, HIV infection, cirrhosis, neoplasia and organ transplant [4–7].

Probiotics are generally considered safe. Rare infections involving lactobacilli or bifidobacteria have been reported; however, the widespread use of probiotics in the general population did not seem to determine an increase of infections [8].

Hereditary hemorrhagic telangiectasia (HHT) or Rendu-Osler-Weber disease is an autosomal dominant disorder characterized by cutaneous telangiectasia, recurrent epistaxis and visceral arteriovenous malformations (AVMs) of the lungs, gastrointestinal tract, liver, and brain. Because AVMs allow the direct communication between pulmonary and systemic circulation, patients with HHT are at higher risk for severe infections like cerebral abscesses, septicemia, arthritis and osteomyelitis; among extracerebral infections, endocarditis is very seldom reported. [9].

Hereby we report a case of infective endocarditis caused by *Lactobacillus rhamnosus* in a patient with HHT who was also a heavy consumer of probiotics, and a review of the literature on similar cases. To our knowledge, this was the first case of infective endocarditis caused by a probiotic strain in a patient with HHT.

## Case presentation

A patient aged > 65 years, affected by HHT, was admitted in March, 2017 to our Institute because of prolonged fever not responding to antibiotics. The patient was the first of 4 siblings; two of them were also affected by HHT. In 2010 after a screening visit in a reference center for HHT a screening for AVMs of the liver, lung and brain was made with negative results. Apart from repeated episodes of epistaxis, often requiring specialist ear, nose and throat (ENT) intervention, nasal packings and laser coagulation, the patient’s medical history was remarkable for repeated infections that required hospitalization during the years. In 2011 the patient had a spondylodiscitis with bacteremia caused by *Streptococcus mutans*; in 2012 a febrile enteritis was diagnosed; in 2015 an aortic bioprosthesis was implanted because of aortic insufficiency. In July, 2016 the patient was hospitalized for septic shock caused by *Staphylococcus aureus* bacteremia. After this episode the patient was admitted to an intensive care unit and, subsequently, had a prolonged stay in a long term care facility; during this stay the patient had prolonged alterations in bowel movements with intermittent diarrhea and therefore started to take probiotics. The probiotics included seven different commercial products containing different types of bacteria and yeasts; three of these contained *Lactobacillus rhamnosus*, which was also the last probiotic used by the patient before the recent admission to our hospital. In November, 2016 the patient was transferred from the long term care facility to a medical ward because of bacteremia caused by *Streptococcus mutans*, which responded to a cycle of four weeks of IV antibiotics. During this hospitalization the patient continued to have alteration in bowel movements; repeated stool examinations for *Clostridium difficile*, *Salmonella*, *Shigella* and other enteric pathogens and a colonoscopy were negative. No obvious infectious foci were detected, and in December, 2016 the patient was discharged at home. Soon after discharge the patient was treated for recurrent epistaxis by an ENT specialist who performed laser coagulation of a nasal bleeding site.

In March, 2017, before the admission to our hospital, the patient, who continued to take probiotics, was treated at home by his attending physician with oral ciprofloxacin followed by IM ceftriaxone for a week, because of episodes of fever and night sweats, without improvement of symptoms. After 6 days of antimicrobial treatment, fever and night sweats persisted together with generalized fatigue and malaise and the patient was admitted to our hospital.

At admission the patient was febrile (37.9 °C) and asthenic; pulse rate was 87 bpm; blood pressure was 110/70 mmHg. Skin examination revealed diffuse telangiectasia of the head, trunk and arms but was negative for signs of embolic phenomena. Cardiac examination revealed a 3/6 diastolic murmur. C reactive protein was elevated at 7.46 mg/dL (normal upper value < 1); erythrocyte sedimentation rate was 100 mm/h; white blood cells were 4860/μl (neutrophils 72.5%, lymphocytes 16%); hemoglobin was 9.0 g/dL and platelets were 218,000/μl; albumin was 3.2 g/dL; blood chemistry, prothrombin time and liver enzymes were within the normal range. Procalcitonin was 0.08 ng/mL, within the normal values. Serology for *Salmonella*, *Brucella*, *Coxiella burnetii*, *Mycoplasma*, *Legionella* was negative. Urine culture was also negative.

After three days of interruption of all antibiotics, three sets of blood cultures were drawn. A transesophageal echocardiogram reported an 11-mm endocarditic vegetation on the cuspids of the biologic prosthesis. According to Duke’s criteria [10], a diagnosis of infective endocarditis was made. Empiric treatment with ceftriaxone 2 g/day and gentamicin 3 mg/kg/day, both by IV route, was started. Five blood cultures yielded gram positive rods, that were identified as *Lactobacillus rhamnosus*. This organism was sensitive to amoxicillin/clavulanate and gentamicin but resistant to ceftriaxone; it was also resistant to penicillin, vancomycin, meropenem, rifampin and trimethoprim/sulphamethoxazole (see Additional file 1). The treatment was then modified; the patient was administered IV amoxicillin/clavulanate 2.2 g q8h plus IV gentamicin 3 mg/kg/day.

The clinical course was unremarkable with progressive clinical improvement. The patient tolerated well antibiotic treatment. Renal functionality remained normal in spite of prolonged administration of gentamicin. Repeat blood cultures performed during hospitalization were negative. Imaging of chest, abdomen, brain and spine showed no signs of embolization. A transesophageal echocardiogram performed after 5 weeks of treatment showed disappearance of the vegetation and a functioning prosthesis. The patient was discharged after a total of 6 weeks of treatment in good clinical conditions. At 6-month follow-up the patient was still in good health with no clinical and laboratory signs of infection.

Case reports of infective endocarditis presumably linked to consumption of probiotics, and of infective endocarditis in patients with HHT, reported between 1 January 1997 and 31 August 2017 were identified through a Medline search. We used the search terms “endocarditis”, “probiotics”, “*Lactobacillus*”, “hereditary hemorrhagic telangiectasia” and “Rendu-Osler”, and limited the search to publications in English, French and Spanish. Additional cases were identified from the references of the retrieved articles.

## Methods

### Systematic review of the literature

We found a total of 10 cases of infective endocarditis apparently linked to previous use of probiotics. We retrieved only 6 cases of infective endocarditis in patients with HHT. The results are shown in Tables 1 and 2, respectively [11–26].

**Table 1:** Infective endocarditis apparently linked to previous use of probiotics

Age, sex	Cardiac valve	Etiology	Probiotics	Risk factors for bacteremia	Treatment (duration weeks)	Outcome	Notes	Ref
67 M	MN	*L.rhamnosus*	1-2 capsules daily of a freeze dried probiotic preparation, each capsule of which containing 2 x 10 ^9 *L. rhamnosus* and several other bacterial species (including *L. acidophilus* and *Streptococcus faecalis*)	Extraction of carious teeth	Ampicillin plus gentamicin (2 w) followed by oral pivampicillin plus probenecid (6 w)	Success (alive at 3 months follow-up)	The authors recommend that patients who are immunosuppressed or have preexisting heart valve disease should avoid probiotic preparations containing *L.rhamnosus*	11
65 M	AN	*L.rhamnosus*	Heavy daily consumption of dairy products, not otherwise specified	Colonoscopy	Ceftriaxone, clindamycin and ciprofloxacin	Success (alive at 18 months follow-up)	The authors do not comment on the consumption of probiotics, while consider colonoscopy as the risk factor for the development of infective endocarditis and the intestine as a portal of entry in this patient	12
23 M	AN (bicuspid)	*L.rhamnosus*	Consumption of up to 1.5 l of yoghurt and sour milk per day	--	Emergency valve replacement; amoxicillin-clavulanate; then penicillin G (6 w)	Success (alive at 12 months follow-up)	The isolates from the patient and the yoghurt had identical biofermentation patterns. However, further typing with the Biolog system (an identification system that uses >100 biochemical reactions) and RAPD-PCR revealed that the pathogen and the yogurt isolate were not identical	13
53 M	AN (rheumatic fever in history)	*L. casei*	Reported consumption of several yogurts per day	Recent dental extraction	Doxycycline plus gentamicin; piperacillin-tazobactam; imipenem; valve replacement surgery	Success	According to the authors although no direct link for the development of endocarditis by *L. casei* and yogurt ingestion could be established, the past history of rheumatic fever could be responsible for a locus of minor resistance in the aortic valve, allowing the establishment of the bacteria and the development of the endocarditis	14
<1 M	CVC, right atrium	*Lactobacillus* spp.	Enteral administration of probiotic containing *Lactobacillus* GG, 3 weeks before symptoms	Alteration in gut mucosal integrity	Penicillin G plus gentamicin (6 w)	Success	*Lactobacillus* isolates from the available blood cultures and the probiotic capsules were analyzed with repetitive element sequence based polymerase chain reaction DNA fingerprinting. The isolates appeared indistinguishable from one another. According to the authors, in this patient recent enteral administration of *Lactobacillus* GG seemed to be the only plausible portal of entry.	15
24 F	AP	*L.rhamnosus*	A preparation of probiotics containing *L.rhamnosus* together with antibiotics 6 weeks before surgery	Alteration in gut mucosal integrity	Unspecified antibiotic treatment	Success	Strain isolated from blood culture showed identical pulsed-field gel electrophoresis profiles to those of the *L.rhamnosus* strains contained in the probiotic. According to the authors this infection was most likely caused by bacterial translocation through a weakened intestinal barrier, possibly linked with ischaemia resulting from the patient’s heart failure, and in the authors’ opinion this report highlights the potential adverse effects of administering probiotics to patients who are presenting with organ dysfunction or failure	16
77 M	MP	*L.paracasei*	Daily consumer of unspecified probiotics	Colonoscopy	Amoxicillin plus gentamicin (2 w) followed by amoxicillin (4 w); valvular repair	Success (alive at 2 months follow-up)	In the authors’ words, this case may highlight the risk of probiotic use in some specific cases (colonoscopy, digestive disease); they also recommend to stop probiotics before digestive surgery or colonoscopy.	17
78 M	AN (bicuspid)	*L.paracasei*	Daily consumer of unspecified probiotics	Dental extraction 6 months before admission	Intravenous clindamycin; surgery	Success	According to the authors this case report may highlight the risk of probiotic use especially in patients with valvular heart disease	18
80 M	AN+MN	*L.rhamnosus*	Daily consumer of yoghurt containing *L.rhamnosus*	Upper endoscopy 1 week before symptoms	Ampicillin plus gentamicin (2w); then penicillin (8w); aortic and mitral valve replacement	Success	The valve and yoghurt *L.rhamnosus* strains were 99.6% identical The yoghurt and blood *L.rhamnosus* isolates had identical bands on pulsed-field gel electrophoresis, but with a 2-band difference so according to the authors the relationship between the isolated organism and the diet is uncertain.	19
28 M	AN (bicuspid)	*L.rhamnosus*	Consumption of approximately 200 ml/ day of commercial dairy probiotic preparations containing *Lactobacillus* spp	---	Ampicillin (6w) plus gentamicin (1w)	Success	According to the authors in this patient consumption of probiotics enriched with lactobacilli could have triggered a mechanism of bacterial translocation from the digestive tract	20

AN= aortic, native; AP = aortic, prosthetic; MN = mitral, native; MP = mitral, prosthetic, CVC = central venous catheter, RAPD-PCR = randomly amplified polymorphic DNA polymerase chain reaction

**Table 2:** Infective endocarditis in patients with HHT

Age, sex	Cardiac valve	Etiology	Type of AV malformations	Treatment (duration days/weeks)	Outcome	Notes	Ref
62 F	MP	*S.mitis*	Skin, nose and mouth TA	Piperacillin + cefazolin (32 d); valve replacement	Success	No AVMs in the lung, brain, liver, gastrointestinal tract or urinary bladder. The patient reported frequent episodes of epistaxis	21
79 F	MN	MSSA	Nose TA, hepatic AVMs	Oxacillin (4 w) + gentamicin (5 d); valve replacement	Dead	Nasal packing for epistaxis reported as a likely portal of entry; the authors propose that in patients with HHT treatment of nasal carriage of *S.aureus* with mupirocine is proposed	22
73 M	AP	No isolation from blood/valve tissue	Nose TA	Unspecified broad-spectrum antibiotics; valve replacement	Success		23
61 F	AN; AP	MRSA	Nose TA, pulmonary/hepatic AVMs	Unspecified antibiotics; emergency surgery; reoperation (Bentall operation)	Relapse, then success	Cardiogenic shock; Relapse of endocarditis on the prosthetic aortic valve; success after reoperation (Bentall operation) and antibiotics The authors define the pulmonary AVMs of this patient at high risk for infection (they were treated with coil embolization)	24
65 F	AP	*S.epidermidis*	Nose TA, pulmonary AVMs	Unspecified antibiotics (>6 w); complex surgery	Success (alive at 9 month follow-up)	The authors propose that PVE in this patient resulted from her anterior nasal packing for recurrent epistaxis with bacteria not trapped because of the pulmonary AVMs. They conclude that recurrent epistaxis may increase the risk of IE, and that patients with HHT and recurrent epistaxis require long-term follow-up.	25
65 M	PN	*S.epidermidis*	Nose TA, hepatic AVMs	Rifampin + linezolid (4 w) then linezolid (2 w); surgery (valvuloplasty)	Success (alive at 2 years follow-up)	The authors state that the infection probably came from the nasal mucosa	26

AN= aortic, native; AP = aortic, prosthetic; MN = mitral, native; PN = pulmonary, native

MRSA = methicillin-resistant *Staphylococcus aureus*; MSSA = methicillin-sensitive *Staphylococcus aureus*;

TA = telangiectasias; AVMs = arteriovenous malformations

PVE = prosthetic valve endocarditis

## Discussion

Endocarditis caused by probiotics and in patients with HHT are both rare conditions and the case we present is likely the result of different pathogenetic mechanisms operating in a single patient.

In cases of sepsis or bacteremia caused by microorganisms considered probiotics, mechanisms like bacterial translocation from the gut or other alterations in the integrity of mucosal surfaces permitting passage of microbes in the bloodstream have been hypothesized. In a series of 89 episodes of bacteremia caused by lactobacilli, the majority of patients had severe underlying diseases, with a predominance of gastrointestinal or hepatic neoplasia, and the mortality at 1 year was high (69%). In this series, *L. rhamnosus* was the most frequent isolate (53%); no cases of endocarditis were found [27]. In a series of 73 endocarditis caused by lactobacilli, 63% of patients had an underlying structural heart disease and 12% suffered from previous episodes of endocarditis. Dental procedures were reported in 47% of cases, but consumption of probiotics was reported only in 3 cases [28].

In our literature search of endocarditis associated with probiotics use, we noticed that all patients had infections caused by microorganisms belonging to the species *Lactobacillus*. The majority of patients were not immunosuppressed, but instead had predisposing factors for bacteremia, like alteration in gut mucosal integrity, colonoscopy or teeth extraction. It is well known that in such circumstances patient may develop bacteremia and endocarditis caused by commensal microorganisms like oral streptococci or saprophytic microorganisms of the gut. The preponderance of *Lactobacillus* among infections caused by probiotics is interesting. It has been shown that *Lactobacillus rhamnosus* strains isolated from endocarditis possess the ability to aggregate platelets, to bind collagen and fibrinogen, and to produce glycosidases and proteases [29]. In an experimental study using the rat model of experimental endocarditis aimed at assessing the potential pathogenicity of probiotic and clinical isolates of *Lactobacillus rhamnosus* and *Lactobacillus paracasei*, it has been showed that at least some of the probiotic strains of *L. rhamnosus* exibit a 90% infective dose (ID_90_) that is comparable to that of clinical isolates of *Lactobacillus*. These probiotic strains share the same fluorescent amplified fragment length polymorphism (FAFLP) as the clinical isolates with the lowest ID_90_, although they can be differentiated from clinical isolates by pulsed-field gel electrophoresis (PFGE) fingerprinting [30]. Moreover, strains of *L. rhamnosus* isolated in endocarditis and other serious infections that were indistinguishable from strains of probiotic *L. rhamnosus* by PFGE have been described [15, 31]. These results suggest that at least in some circumstances probiotic strains of *L. rhamnosus* can give rise to bacteremia and endocarditis. Lactobacilli are intrinsically resistant to vancomycin, and may be resistant to various other antibiotics [32], so the treatment of infections caused by these microorganisms can be difficult.

In a series of 85 blood isolates of *Lactobacillus* [33] all species demonstrated low minimum inhibitory concentrations (MICs) of imipenem, piperacillin-tazobactam, netilmicin and clindamycin and high MICs of vancomycin; instead, the MICs of benzilpenicillin, ampicillin, ceftriaxone and other cephalosporins varied widely among species and among different isolates of the same species. Among cephalosporins, ceftriaxone showed the highest MIC values for isolates of *L. rhamnosus*. Ampicillin, broad-spectrum cephalosporins and vancomycin are often used in empiric treatment of endocarditis, but they could prove ineffective in bloodstream infection caused by lactobacilli.

People with HHT are predisposed to infections, because of the presence of pulmonary, hepatic and gut arterovenous malformation. The fragile mucosa of the nasal cavity is also considered a portal of entry for pathogenic microorganisms in these patients. The majority of infections reported in HHT patients are cerebral abscesses, however other infections like hepatic abscesses, bacteremia, septic arthritis, osteomyelitis, skin infections and infective endocarditis are also reported [10, 34]. In our literature search, we found only 6 cases of infective endocarditis in patients with HHT, caused by coagulase positive and negative staphylococci and *S. mitis*. The mortality rate for these episodes was 16%. The causative microorganisms were common commensal of the skin, nose, mouth, oropharyngeal tract and gut. The majority of the patients reported frequent epistaxis with nasal packing but no other risk factors for bacteremia. It has been hypothesized that nasal mucosa may be the portal of entry for microorganisms in case of nasal trauma associated with nasal packing; in other cases, proliferation of microorganisms like *S. aureus* was favored by the presence of foreign material in the nasal cavity [35, 36]. It has been suggested that patients with HHT are considered at risk of bacteremia and endocarditis after dental procedures, and should be therefore treated with prophylactic antibiotics in these circumstances [37]; the same risk could be assumed for nasal procedures involving trauma to the nasal mucosa, and in these circumstances, antibiotic prophylaxis should probably be offered in these patients. It has also been suggested that these patient undergo nasal decontamination with mupirocin before nasal packing [38].

In the case of our patient, probably different factors have concurred to the occurrence of endocarditis. Although the patient’s history of recurrent infections and bacteremia can raise the suspect of an immune depression, this was not apparently the case. The levels of lymphocytes, neutrophils, immunoglobulins and complement were normal during intervals free of disease. Human immunodeficiency virus (HIV) serology was negative; the patient had no signs of neoplasia and was not taking immunosuppressive drugs. In the patient’s previous infective episodes, the isolates (*Streptococcus mitis*, *Staphylococcus aureus*) were typical pathogenic strains. Although the portal of entry of these bacteria was not clear, as apparently the patient had not an arteriovenous malformation in the lung, gut or brain, we hypothesize that the portal of entry was one of the numerous arteriovenous malformation in the nose or the oral cavity.

During the last infectious episode, the patient underwent a prolonged hospitalization in an intensive care unit and then in a long-term facility, received many courses of antibiotics and suffered from prolonged episodes of diarrhea; during this period the patient started to take large amounts of probiotics, many of them containing lactobacilli; therefore, it is plausible to assume that an imbalance in the bacterial flora of the gut and/or the oral cavity, and perhaps the nose, with a relative predominance of *Lactobacillus* could have occurred. We were not able to characterize the lactobacilli contained in the probiotics ingested by the patient, so we can only suppose that this strain of *Lactobacillus rhamnosus* was of dietary origin (we recognize that this is a limitation of our study). During the months before the last infectious episode, the patient had many episodes of prolonged epistaxis and underwent many traumatic nasal interventions such as nasal packing and cauterization of nasal telangiectasias. In patients with HHT, prolonged epistaxis is associated with extracerebral infections [9] and nasal instrumentation has been suggested as a possible risk factor for bacteremia [35]. Moreover, we cannot exclude other mechanisms and portals of entry of microbes in the bloodstream, such as microbial translocations of bacteria from the gut. Finally, the presence of a valvular aortic prosthesis was a clear predisposing factor for the development of infective endocarditis.

## Conclusions

In conclusion, consumption of probiotics can pose a risk of serious infections in patients with particular predisposing factors to infections such as patients with HHT. These patients and their caregivers should be aware of the increased risk of infective endocarditis, especially during prolonged episodes of nasal bleeding and after nasal packing or other traumatic interventions in the nose. When these conditions are present, in our opinion antibiotic prophylaxis should be considered.

## Additional file

Additional file 1: Microbiology. Description of methods used to identify the microorganism isolated from blood culture, both by standard microbiology culture and by genetic analysis; minimum inhibitory concentrations (MICs) of the antibiotics used for testing. (DOCX 11 kb)

## Abbreviations

AN: Aortic, native
AP: Aortic, prosthetic
AVMs: Arteriovenous malformations
CVC: Central venous catheter
ENT: Ear, nose and throat
FAFLP: Fluorescent amplified fragment length polymorphism
HHT: Hereditary hemorrhagic telangiectasia
HIV: Human immunodeficiency virus
ID_90_: 90% infective dose
IE: Infective endocarditis
IM: Intramuscular
IV: Intravenous
MICs: Minimum inhibitory concentrations
MN: Mitral, native
MP: Mitral, prosthetic
MRSA: Methicillin-resistant *Staphylococcus aureus*
MSSA: Methicillin-sensitive *Staphylococcus aureus*
PFGE: Pulsed-field gel electrophoresis
PN: Pulmonary, native
PVE: Prosthetic valve endocarditis
RAPD-PCR: Randomly amplified polymorphic DNA polymerase chain reaction
TA: Telangiectasias
