# Supplementary material for: Probiotics and infective endocarditis in patients with hereditary hemorrhagic telangiectasia: a clinical case and a review of the literature
Source: BMC Infect Dis. 2018 Feb 1;18:65. doi: 10.1186/s12879-018-2956-5 (PMC5796351; doi:10.1186/s12879-018-2956-5)
Supplement: Additional file 1: — Microbiology. Description of methods used to identify the microorganism isolated from blood culture, both by standard microbiology culture and by genetic analysis; minimum inhibitory concentrations (MICs) of the antibiotics used for testing. (DOCX 11 kb) [file 12879_2018_2956_MOESM1_ESM.docx]

Additional file. Microbiology

Species identification was performed using matrix-assisted laser desorption/ionization time-of-flight (Maldi Tof) mass spectrometry (Brucker Daltonic GmbH, Germany) and confirmed by whole-genome sequencing (WGS).

Antimicrobial susceptibility testing was performed using E-test strips (Liofilchem, Italy) on selective medium for lactobacilli Rogosa agar plates (Liofilchem, Italy) (Table 1).

Minimum inhibitory concentrations (MICs) were interpreted according to the European Committee on Antimicrobial Susceptibility Testing (EUCAST) recommendations.

Susceptibility testing for aminoglycosides was performed using disk diffusion.

Table 1. Summary of the susceptibility pattern of the clinical *Lactobacillus rhamnosus* isolate

| Antibiotic | MIC (mg/L) |
| --- | --- |
| Vancomycin | ≥256 |
| Tetracycline | 0.75 |
| Erythromycin | 0.25 |
| Levofloxacin | 1 |
| Moxifloxacin | 0.19 |
| Daptomycin | 1 |
| Clindamycin | 0.125 |
| Rifampicin | ≥32 |
| Ciprofloxacin | 1.5 |
| Chloramphenicol | 4 |
| Trimethoprim/sulfamethoxazole | ≥32 |
| Piperacillin-tazobactam | ≥256 |
| Cefotaxime | 16 |
| Amoxicillin/clavulanic acid | 2 |
| Ertapenem | 8 |
| Imipenem | ≥32 |
| Meropenem | ≥32 |
| Tigecycline | 0.032 |

Minimal Inhibitory Concentrations (MIC) were performed using E-test method and interpreted according to the European Committee on Antimicrobial Susceptibility Testing (EUCAST) criteria.

Sequence data for analysis was generated by WGS at the Italian Institute for Technologies (ITT), Rome Italy. DNA was extracted and library were prepared with a Nextera XT DNA sample preparation kit (Illumina, San Diego, CA), according to the manufacturer’s instructions. Complete genome sequence was obtained using the Illumina MiSeq next generation sequencer with 2x250PE.

The nucleotide sequence was analysed at the NCBI website (http://www.ncbi.nlm.nih.gov) by the Basic Local Alignment Search Tool (BLAST) programme and showed 98% nucleotide identity with the genome of *L. rhamnosus* ATCC 53103 strain (Genbank accession no. AP011548.1). ResFinder software was used to identify antibiotic resistance genes; no resistant determinants were detected.
